# Characterization of *Pestivirus tauri* (BVDV-2, Subtype c) Isolates in Northern Italy Using Whole-Genome Sequencing

**DOI:** 10.3390/v18030367

**Published:** 2026-03-16

**Authors:** Enrica Sozzi, Maya Carrera, Chiara Chiapponi, Laura Soliani, Ambra Nucci, Rita Muratore, Gabriele Leo, Anna Marelli, Davide Lelli, Tiziana Trogu, Clara Tolini, Giovanni Loris Alborali, Moira Bazzucchi, Ana Moreno

**Affiliations:** Istituto Zooprofilattico Sperimentale della Lombardia e dell’Emilia Romagna “Bruno Ubertini” (IZSLER), Via Antonio Bianchi 7/9, 25124 Brescia, Italy; maya.carrera@izsler.it (M.C.); chiara.chiapponi@izsler.it (C.C.); laura.soliani@izsler.it (L.S.); ambra.nucci@izsler.it (A.N.); rita.muratore@izsler.it (R.M.); gabriele.leo@izsler.it (G.L.); anna.marelli@izsler.it (A.M.); davide.lelli@izsler.it (D.L.); tiziana.trogu@izsler.it (T.T.); clara.tolini@izsler.it (C.T.); giovanni.alborali@izsler.it (G.L.A.); moira.bazzucchi@izsler.it (M.B.); anamaria.morenomartin@izsler.it (A.M.)

**Keywords:** *Pestivirus tauri*, subtype 2c, whole genome sequencing, cattle, Northern Italy

## Abstract

Bovine viral diarrhea (BVD) is a major cause of economic losses in the global cattle industry, particularly in countries characterized by intensive livestock production systems. *Pestivirus tauri*, formerly known as Bovine viral diarrhea virus type 2 (BVDV-2), is the current taxonomic designation according to the International Committee on Taxonomy of Viruses (ICTV). Between 2005 and 2018, *Pestivirus tauri* was detected in cattle herds in mainland Italy, particularly in the Lombardy region. Four viral strains were successfully isolated in cell cultures and subjected to whole-genome sequencing. Phylogenetic reconstruction placed all Italian isolates within the *Pestivirus tauri* subgenotype c, a lineage encompassing strains reported in Asia, Europe and the United States. Consistently, comparative sequence identity analyses indicated the highest similarity with the Parker strain (USA, 1991) and the Potsdam 1600 strain (Germany, 2000). These results contribute to a more detailed understanding of *Pestivirus tauri* genomic architecture and evolutionary dynamics, providing a valuable resource for comparative genomic studies. Such data are crucial for exploring viral diversity and evolution, optimizing the design of diagnostic primers and probes, and advancing insights into the molecular epidemiology of *Pestivirus*.

## 1. Introduction

Bovine *Pestivirus* infections are recognized as significant viral diseases in cattle herds worldwide, with severe economic and sanitary repercussions [1,2]. *Pestivirus* genus belongs to the *Flaviviridae* family, which also comprises the *Orthoflavivirus*, *Hepacivirus*, and *Pegivirus* genera [3]. According to the latest taxonomic release of the International Committee on Taxonomy of Viruses (ICTV) (July 2022; amended March 2023, MSL #38), the genus *Pestivirus* currently comprises 19 species [4]. More recently, the ICTV *Flaviviridae* Study Group proposed revisions to the species names within the family *Flaviviridae*, following the ICTV decision to adopt a standardized binomial nomenclature for all established species [4]. However, only eleven established species have been assigned binomial names, whereas the remaining eight are still designated as *Pestivirus L* to *Pestivirus S* (Table 1).

Bovine *Pestiviruses* include the species *Pestivirus bovis* (formerly *Pestivirus A*; bovine viral diarrhoea virus-1; BVDV-1), *Pestivirus tauri* (*Pestivirus B*; BVDV-2), and *Pestivirus brazilense* (*Pestivirus H*; HoBiPeV) [4,5,6]. These species have been further classified into 23 (a–x), 5 (a–e), and 5 (a–e) subgenotypes, respectively, based on sequence analysis of the 5′ untranslated region (5′-UTR), N^pro^, and E2 genomic regions [7,8,9,10]. The *Pestivirus* genome consists of a single-stranded positive-sense RNA of approximately 12.3 kb in length [11]. A single open reading frame encodes four structural proteins (C, Erns, E1, and E2) and seven non-structural proteins (Npro, p7, NS2-3, NS4A, NS4B, NS5A, and NS5B) and is flanked by 5′- and 3′-untranslated regions (UTRs) [11]. Bovine *Pestiviruses* cause a substantial economic burden on the cattle industry by inducing respiratory disease, decreasing milk production, causing fetal abortion, leading to the birth of weak and persistently infected (PI) calves, and establishing long-term infections [12]. *Pestivirus* prevalence is highest in cattle-producing countries that lack effective control measures, with persistently infected animals playing a key role in viral maintenance [13]. In Italy, *Pestivirus* has been present since the 1960s, with an increasing seroprevalence in dairy herds [14]. Molecular studies revealed high genetic variability among circulating Italian strains [15,16,17,18,19]. A compulsory program is ongoing in Bolzano and Trentino provinces, whereas voluntary control programs are applied in few other Northern regions (Piedmont, Veneto, Friuli-Venezia Giulia) [20]. Different vaccines are commercially available, developed for use in both beef and dairy cattle [21].

*Pestivirus tauri* was first identified in the USA [22,23] and then detected in several countries [24,25,26,27]. Contaminated fetal bovine serum or other biological products likely contributed to the introduction of *Pestivirus tauri* into Europe [28], where it circulates at lower rates than *Pestivirus bovis* [5]. In Italy, *Pestivirus tauri* has been reported in both cattle [29] and small ruminants since the 1990s [30]. Despite its early identification in our country, *Pestivirus tauri* has occurred only sporadically in cattle [31,32,33,34], with 2a being the most prevalent subgenotype in Italy [14], as well as globally [7]. More recently, 2c strains have been detected in Southern Italy in cattle and, to an even greater extent, in small ruminants [32].

Given its increasing relevance in the last decade, studying the full-length genomic sequences of *Pestivirus tauri* is crucial for understanding its genetic diversity, evolution, and epidemiology. Such information can be useful in the development of more effective diagnostic tools to monitor the circulation of the virus. The present study reports determination and characterization of the full-length genomic sequences of *Pestivirus tauri* strains which were isolated from four cattle in Northern Italy.

## 2. Materials and Methods

### 2.1. Sample Collection and Virus Isolation

Between 2005 and 2018, within the framework of epidemiological surveillance programs on *Pestiviruses* funded by the Italian Ministry of Health, four *Pestivirus tauri* were successfully isolated. *Pestivirus tauri* strains were isolated from clinical samples using standard virus isolation techniques. Briefly, serum samples or tissue homogenates obtained at necropsy were inoculated onto confluent monolayers of bovine primary kidney cells or Madin–Darby bovine kidney (MDBK) cells, previously confirmed to be free of contamination with endogenous *Pestiviruses*. Minimal Essential Medium (MEM; Thermo Fischer Scientific, Waltham, MA, USA) was supplemented with gamma ray–irradiated fetal bovine serum, free of *Pestivirus* antigens, genomes, and antibodies. The infected cells were incubated at 37 °C with 5% CO_2_ and checked daily for cytopathic effects (CPEs). Two blind passages were carried out, each following 6–7 days of incubation. Each passage of the inoculated cell culture monolayer was tested using a homemade ELISA virologic screening assay based on monoclonal antibodies. Incremental increases in optical density (OD) were used to confirm viral isolation. Isolated samples were obtained from cattle farms in the Lombardy region of Northern Italy, as follows:

- 2005, strain 206041 was isolated from a visceral pool of a heifer;
- 2015, strain 277457-2 was isolated from a blood sample of a persistently infected animal;
- 2016, strain 169121 was isolated from a visceral pool of a three-month-old calf presenting with necrotic-hemorrhagic enteritis, involving Peyer’s patches and inflamed gingival margins;
- 2018, strain 64643 was isolated from a visceral pool of a two-month-old calf.

### 2.2. Viral RNA Extraction and Real-Time RT-PCR for Pestivirus Screening and Typing

Serum samples and tissue homogenates were subjected to centrifugation at 3000× *g* for 15 min, after which RNA was extracted using the BioSprint 96 One-For-All Vet Kit (Qiagen, Hilden, Germany) on a KingFisher^®^ Apex Purification System (Thermo Fisher Scientific, Waltham, MA, USA), according to the manufacturers’ instructions. *Pestiviruses* were detected using a real-time RT-PCR screening method, following the procedure previously described by Hoffmann et al. [35]. This method is considered a pan-*Pestivirus* assay, as it is routinely used to detect the most relevant species of the genus (*Pestivirus bovis*, *Pestivirus tauri*, *Pestivirus suis*, *Pestivirus ovis*). Positive samples were subsequently characterised following a protocol described previously [36].

### 2.3. Whole-Genome Sequencing

The complete genomes of the viral strains isolated on cell culture were obtained using the MiSeq^TM^ platform (Illumina, Inc., San Diego, CA, USA). Sequencing libraries were made with an Illumina TruSeq RNA Sample Preparation Kit v 2 (Illumina, Inc., San Diego, CA, USA) according to the manufacturers’ instructions. Following sequencing, raw reads of *Pestivirus tauri* were de novo assembled using CLC Genomic Workbench v.11 (QIAGEN, Milan, Italy) with an average coverage of 1468×. The resulting assembled sequences were queried with BLAST version 2.16.0 (NCBI, Bethesda, MD, USA) to identify the closest *Pestivirus tauri* strain, which were subsequently used in the reference-based mapping step, to obtain the final consensus sequences.

### 2.4. Phylogenetic and Bioinformatics Analysis

The complete genome nucleotide sequences of *Pestivirus tauri* strains were aligned and compared with several sequences available in GenBank (www.ncbi.nlm.nih.gov; accessed on 16 June 2025). Partial 5′ UTR sequences of *Pestivirus tauri* strains were retrieved from public databases to investigate the phylogenetic relationships of circulating strains. Two datasets were generated to maximize sequence inclusion. The first dataset comprised a shorter fragment (positions 164–357 of the reference genome) to include sequences from Italy and neighboring countries. The second dataset included a longer fragment (positions 89–420), encompassing the 5′ UTR and the initial portion of the N^pro^ coding region, allowing the inclusion of sequences from a broader global collection. Selected sequences were aligned using MAFFT v7.525 [37]. Phylogenetic trees were inferred with IQ-TREE v3.0.1 [38] using 1000 bootstrap replicates, applying the best-fit substitution model automatically selected by the software. Tree visualization was carried out using FigTree v1.4.4 [39]. Nucleotide identity of the 2018 PX121312 sequence was calculated against 53 *Pestivirus tauri* sequences using a custom Python script (Python 3.7.15). For the polyprotein coding region, position-specific identity was computed with Biopython v1.79 [40], excluding sequences which determined gaps in the alignment. At each genomic position, the proportion of sequences carrying the most frequent nucleotide was determined. For each position *i*, identity was defined as:

**Identity (*i*) = (frequency of the most common nucleotide at position *i*)/(number of nucleotides at position *i*)**

The resulting identity values were exported as a table and visualized across the genome as a bar plot using Matplotlib (v3.5.3) [41].

### 2.5. Nucleotide Sequence Accession Numbers

The whole-genome sequences of *Pestivirus tauri* strains obtained in this study were submitted to GenBank (NCBI) under accession numbers PX121309 to PX121312.

## 3. Results

**Figure 1:**
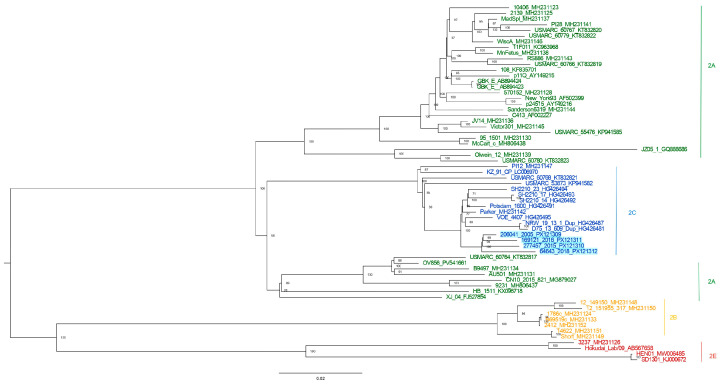
Maximum-likelihood phylogenetic tree of *Pestivirus tauri* complete genome sequences. Tree branches are colored according to subtype: 2a (green), 2c (blue), 2b (yellow), and 2e (red). Bootstrap values (1000 replicates) are shown. The accession number used were the following: PX121312, PX121310, PX121311, PX121309, MH231123, KF835701, KC963968, MH231148, MH231150, MH231124, MH231151, MH231125, MH231152, MH231126, MH231128, MH806437, MH231130, MH231131, MH231133, MH231134, AF002227, MG879027, HG426481, AB894424, AB894423, KX096718, MW006485, AB567658, MH231136, GQ888686, MH806438, LC006970, AF502399, AY149216, MH231137, MH231138, HG426487, MH231139, AY149215, MH231142, MH231147, MH231141, HG426491, MH231143, MH231144, KJ000672, HG426492, HG426493, HG426494, MH231149, KP941582, KP941585, KT832817, KT832819, KT832820, KT832821, KT832822, KT832823, MH231145, HG426495, MH231146, FJ527854.

Over the 2005–2018 study period, four *Pestivirus tauri* strains were successfully isolated. Positive samples originated from intensive cattle farms in the Lombardy region, specifically in the provinces of Mantua (*n* = 3) and Lodi (*n* = 1). All samples tested positive by real-time RT-PCR screening and were subsequently genotyped. Virus isolation was performed on MDBK cell lines, and all four strains belonged to the non-cytopathic biotype. Whole-genome sequencing was performed on the isolates using the Illumina platform. Phylogenetic analysis of the four Italian strains showed that they cluster together on a distinct branch within the same clade, closely related to subtype c (Figure 1). The complete genomes exhibited the highest nucleotide identity with two previously characterized strains: the Parker strain, isolated in the USA in 1991 [42], and the Potsdam 1600 strain, identified in Germany in 2000 [43]. Phylogenetic analysis of the longer fragment spanning the partial 5′ UTR and the initial portion of the N^pro^ coding region revealed a different clustering pattern. In this tree, the strains collected in 2005 and 2015 grouped together in a distinct cluster, whereas the 2016 strain clustered with the US strain USMARC_53873_(KP941582, 2014) and the 2018 strain grouped with VOE_4407 strain (HG426495, 2007) isolated in Germany_2007 (Supplementary Materials, Figure S1).

In contrast, phylogenetic analysis based on the shorter 5′ UTR fragment, including Italian sequences and those from neighboring countries, showed that—apart from a few exceptions (AJ293603.1, Italy 1997, and EU327594.1, Austria 2003), which formed a separate cluster—isolates generally tended to group according to their country of origin, suggesting a geographic structuring of the circulating strains (Figure S2).

**Figure 2:**
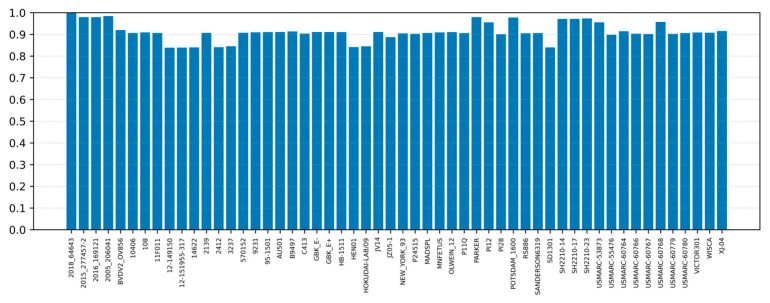
Nucleotide identity (ranging from 0 to 1) between the 2018 reference sequence PX121312 and the sequences included in the phylogenetic analysis. Sequences are identified by strain, with accession numbers listed from left to right: PX121312, PX121310, PX121311, PX121309, PV541661, MH231123, KF835701, KC963968, MH231148, MH231150, MH231151, MH231125, MH231152, MH231126, MH231128, MH806437, MH231130, MH231131, MH231134, AF002227, AB894424, AB894423, KX096718, MW006485, AB567658, MH231136, GQ888686, AF502399, AY149216, MH231137, MH231138, MH231139, AY149215, MH231142, MH231147, MH231141, HG426491, MH231143, MH231144, KJ000672, HG426492, HG426493, HG426494, KP941582, KP941585, KT832817, KT832819, KT832820, KT832821, KT832822, KT832823, MH231145, MH231146, FJ527854.

Sequence identity analysis was performed on 50 genomes retrieved from NCBI, together with all the sequences generated in this study, using the genomic region spanning nucleotide positions from 356 to 11,957 and the 2018 strain PX121312 as reference (Figure 2, Table S1). The lowest nucleotide identity was observed for a 2b strain collected in the United States in 2012 (MH231148), which showed an identity of 0.8386. This was followed by strains isolated in the same year and location, MH231150 (0.8393) and MH231151 (0.8402), as well as by a 2e strain isolated in China in 2012 (KJ000672), which showed a sequence identity of 0.8402.

The highest nucleotide identity was observed for the 2005 strain generated in this study (206041), with an identity value of 0.9842. The second highest identity was shared by the Parker strain and the Italian strain generated in 2016 (169121), both showing a nucleotide identity of 0.9797. This was followed by the 2015 Italian strain (277457-2), which displayed a sequence identity of 0.9793. Among the remaining sequences, the Potsdam strain showed a comparatively high nucleotide identity of 0.9777.

**Figure 3:**
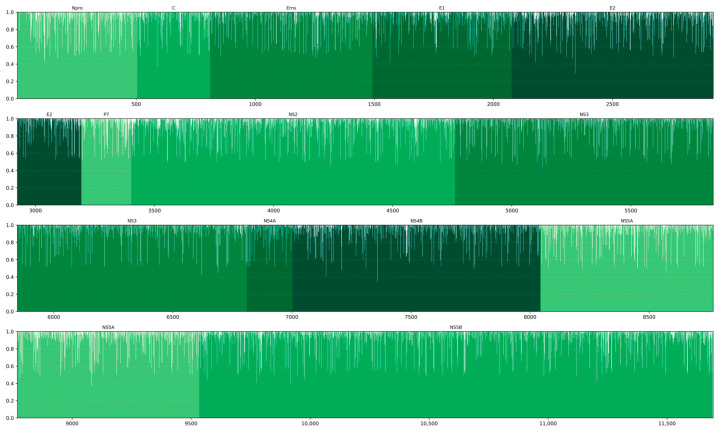
Nucleotide conservation across *Pestivirus tauri* strains. A total of 52 sequences were included in the analysis; coding regions are highlighted according to the protein they translate. The scale on the left represents nucleotide conservation on a 0–1 scale, where a full bar indicates complete conservation. The accession numbers employed in this analysis are the following: PV541661, PX121312, PX121310, PX121311, PX121309, MH231123, KF835701, KC963968, MH231125, MH231126, MH231128, MH806437, MH231130, MH231131, MH231134, AF002227, MG879027, HG426481, AB894424, AB894423, KX096718, MW006485, AB567658, MH231136, GQ888686, MH231137, MH231138, HG426487, MH231139, AY149215, MH231142, MH231147, MH231141, HG426491, MH231143, MH231144, KJ000672, HG426492, HG426493, HG426494, KP941582, KP941585, KT832817, KT832819, KT832820, KT832821, KT832822, KT832823, MH231145, HG426495, MH231146, FJ527854.

All positions of the polyprotein coding sequence were furthermore analyzed, as shown in Figure 3 (Table S2 and S3), in order to identify the most variable regions. A total of 52 sequences were included in the analysis, excluding sequences that generated a gap in the alignment. The analysis was performed on a region-by-region basis within the polyprotein coding sequence, focusing on the sub-regions corresponding to individual mature proteins, with values ranging from 0 (absence of conservation) to 1 (complete conservation). Overall, the lowest level of conservation was observed for the P7 coding region (0.9316), followed by Npro (0.9338) and E2 (0.9376). Intermediate conservation levels were detected for NS2 (0.9383), C (0.9431), NS5A (0.9432) and Erns (0.9434). Higher conservation was observed for E1 (0.9459), NS5B (0.9485), NS4A (0.9496), and NS4B (0.9533), with NS3 displaying the highest conservation among all analyzed proteins (0.9544).

The nucleotide positions exhibiting the lowest degree of conservation were 2343, 7359 and 588, corresponding to the E2, NS4B, and C proteins, respectively. Position 2343 displayed the highest divergence value (0.2885), followed by position 7359 (0.3461) and 588 (0.3654). The nucleotide variation at position 2343 (19.2% T, 26.9% C, 25.0% A, 28.8% G) occurred at the third codon position and resulted in a non-synonymous substitution in the E2 protein, leading to an amino acid change between aspartate (D) and glutamate (E). Similarly, the variation at position 7359 (32.7% T, 32.7% C, 34.6% G) was located at the third codon position and produced a non-synonymous substitution in NS4B, resulting in an amino acid change from methionine (M) to isoleucine (I), which involves a shift from a polar to a non-polar residue. In contrast, the mutation at position 588 (30.8% T, 21.1% C, 36.5% A, 11.5%) occurred at the third codon position and was synonymous, yielding no alteration in the encoded amino acid.

## 4. Discussion

In this study, four *Pestivirus tauri* isolates were successfully obtained from intensive cattle farms in Northern Italy between 2005 and 2018, providing a longitudinal dataset for the region. Positive samples were successfully isolated on MDBK cells and confirmed as non-cytopathic, a biotype which is commonly associated with persistent infection and is a major driver of viral dissemination in cattle populations [44].

Phylogenetic analysis placed the four Italian strains in a distinct cluster within the same clade, which belonged to subtype 2c. This clustering suggests that the strains circulating in Lombardy during the study period may represent a localized lineage, potentially maintained through farm-to-farm transmission. Notably, the high identity values observed among the Italian isolates indicate a relatively slow evolutionary rate in the local population or limited introduction of novel *Pestivirus tauri* lineages into the farms included in the study. The subtype 2c was previously reported in other European countries, such as Germany [24] and United Kingdom [45], in 1996 and 2001, respectively [5]. Remarkably, the highest nucleotide identity across the complete genomes obtained in this study was observed with the Parker strain (USA, 1991) [42] and the Potsdam 1600 strain (Germany, 2000) [43], suggesting that the Italian lineage shares ancestry with strains previously identified in America and Europe. This pattern may reflect historical introductions followed by local evolution, or the presence of conserved lineage-defining genomic features within subtype 2c. Phylogenetic analysis based on a fragment spanning the partial 5′ UTR and the initial portion of the N^pro^ coding region revealed relationships that differed from those inferred from the polyprotein-based phylogeny. While the whole-genome analysis indicated a common clustering of the Italian strains, the 5′ UTR tree separated the isolates into different clusters, with the 2005 and 2015 strains grouping together, whereas the 2016 and 2018 strains clustered with strains isolated from the United States and Germany, respectively. However, the phylogenetic reconstruction based on this region showed limited statistical support, with bootstrap values frequently below 70, particularly at internal nodes, indicating a lower robustness of the inferred relationships. These differences likely reflect the distinct evolutionary constraints acting on different genomic regions. In particular, the 5′ UTR is a non-coding regulatory region and may evolve at a different rate compared with protein-coding regions of the genome, potentially leading to alternative phylogenetic inferences. In contrast, consistent with the whole-genome findings, the phylogenetic pattern inferred from the shorter partial 5′ UTR analysis, which included sequences from Italy and neighboring countries, supports the local circulation of *Pestivirus tauri* strains in this geographic area. In this dataset, isolates generally clustered according to their country of origin, suggesting the persistence of locally maintained viral lineages over time. Nevertheless, AJ293603.1 and EU327594.1 isolated in Italy in 1997 and in Austria in 2003, respectively, formed a distinct cluster, representing notable exceptions to this geographic pattern. These discrepancies may reflect trade-related introductions, or the limited phylogenetic resolution provided by the relatively short 5′ UTR fragment. Nucleotide identity analysis revealed a broad range of genetic divergence, with the lowest identities observed with 2b (MH231148, MH231150, MH231151) and 2e (KJ000672) strains isolated from the U.S. and China, respectively. Interestingly, the 2018 Italian strain showed higher similarity to the 2005 strain than to the 2015 and 2016 isolates, despite their closer temporal proximity. Furthermore, the 2018 Italian strain showed a higher identity to the Parker strain from USA than to the 2015 strain isolated in the same region.

Region-by-region conservation analysis of the polyprotein inferred from the corresponding nucleotide sequence revealed differential variability among structural and non-structural proteins. The lowest conservation was observed in P7, N^pro^, and E2, whereas NS3 was the most conserved protein. The high variability in E2 is expected, as this glycoprotein is the primary target of neutralizing antibodies and is subject to strong immune-driven selection [46]. Similarly, elevated divergence in N^pro^ is consistent with its role in immune evasion through interferon antagonism, where adaptive changes may enhance viral fitness in response to host immune pressure [47]. However, the N-terminal portion of N^pro^ appears to be highly conserved, likely reflecting the presence of key functional domains involved in the regulation of viral translation [48]. Conversely, the high conservation of NS3 aligns with its essential function as a viral helicase/protease complex required for replication, which typically constrains sequence variation [49].

Analysis of the most variable nucleotide positions identified three sites with the highest divergence: 2343 (E2), 7359 (NS4B), and 588 (C). Notably, positions 2343 and 7359 were associated with non-synonymous substitutions. The substitution in E2 (position 2343) involved an aspartate-to-glutamate change, representing a conservative substitution between two acidic residues. The non-synonymous change in NS4B (position 7359) resulted in a methionine-to-isoleucine substitution. Although both amino acids are hydrophobic, this change may alter local protein conformation. In contrast, the synonymous substitution at position 588 suggests that genetic variability in the C protein may be largely neutral, possibly reflecting strong selective constraints at the amino acid level but tolerance for nucleotide changes.

In Italy, vaccination against *Pestivirus tauri* is currently optional, and only a few regions have implemented voluntary virological monitoring to identify persistently infected animals. At the European level, vaccines targeting type 2 have been available since 2015. In Italy, the number of commercially available vaccines specifically targeting *Pestivirus tauri* is limited to subtype 2a. Given that vaccination remains optional and active monitoring is limited, there are currently insufficient data to determine whether these vaccines provide effective protection against subtype 2c strains. Therefore, ongoing monitoring of *Pestivirus tauri* genotype distribution is essential to guide appropriate vaccine selection and to improve the overall effectiveness of control programs.

Overall, the results of this study highlight the persistence of a genetically stable *Pestivirus tauri* lineage in Lombardy over more than a decade, with limited introduction of divergent strains. Future studies should expand sampling to additional regions and incorporate epidemiological data to better reconstruct transmission dynamics, as well as functional assays to determine the phenotypic impact of the identified amino acid substitutions.

## 5. Conclusions

The complete genome sequences of the four *Pestivirus tauri* strains described in this study broaden the available molecular information on full-length *Pestivirus* genomes. These data contribute to a deeper understanding of *Pestivirus* genomic organization and provide a valuable resource for comparative genetic analyses, which are essential for investigating viral evolution and diversity, improving the design of diagnostic primers and probes, and advancing knowledge of *Pestivirus* molecular epidemiology.

## Figures and Tables

**Table 1:** Previous and updated taxonomy of established *Pestivirus* species in accordance with the ICTV-mandated binomial nomenclature.

PREVIOUS TAXONOMY		NEW TAXONOMY
Species	Abbreviation	Species
*Pestivirus A*	BVDV-1	*Pestivirus bovis*
*Pestivirus B*	BVDV-2	*Pestivirus tauri*
*Pestivirus C*	CSFV	*Pestivirus suis*
*Pestivirus D*	BDV	*Pestivirus ovis*
*Pestivirus E*	PHV	*Pestivirus antilocaprae*
*Pestivirus F*	BuPV	*Pestivirus australiaense*
*Pestivirus G*	-	*Pestivirus giraffae*
*Pestivirus H*	HoBi	*Pestivirus brazilense*
*Pestivirus I*	-	*Pestivirus aydinense*
*Pestivirus J*	NrPV	*Pestivirus ratti*
*Pestivirus K*	APPV	*Pestivirus scrofae*
*Pestivirus L*	Linda V	-
*Pestivirus M*	PhoPeV	*-*
*Pestivirus N*	TSV	-
*Pestivirus O*	ovIT PeV	-
*Pestivirus P*	DYPV	-
*Pestivirus Q*	RtNn-PeV	-
*Pestivirus R*	RtAp-PeV	-
*Pestivirus S*	BtSk-PeV	-

## Data Availability

The data presented in this study are available on request. The sequences obtained in this study are available in GenBank under accession numbers.

## Supplementary Materials

The following supporting information can be downloaded at: https://www.mdpi.com/article/10.3390/v18030367/s1, Figure S1: Maximum-likelihood phylogenetic tree based on the partial 5′ UTR region of worldwide strains. Bootstrap analysis was performed with 1000 replicates, with only values ≥ 68 shown. The sequences produced in this study are highlighted in light blue; Figure S2: Maximum-likelihood phylogenetic tree based on the partial 5′ UTR and the first portion of the N^pro^ region of strains from Italy and neighboring countries. Bootstrap analysis was performed with 1000 replicates, and only bootstrap values ≥ 70% are shown. The sequences produced in this study are highlighted in light blue; Table S1: Nucleotide identity values between the 2018 reference sequence PX121312 and the sequences included in Figure 2; Table S2: Nucleotide conservation across *Pestivirus tauri* strains included in Figure 3, showing nucleotide identity values position by position, highlighting the protein they encode; Table S3: Mean nucleotide identity calculated for each coding region employed in the analysis shown in Figure 3.
