# Supplementary figures and images for: Characterization of Pestivirus tauri (BVDV-2, Subtype c) Isolates in Northern Italy Using Whole-Genome Sequencing
Source: Viruses. 2026 Mar 16;18(3):367. doi: 10.3390/v18030367 (PMC13030877; doi:10.3390/v18030367)

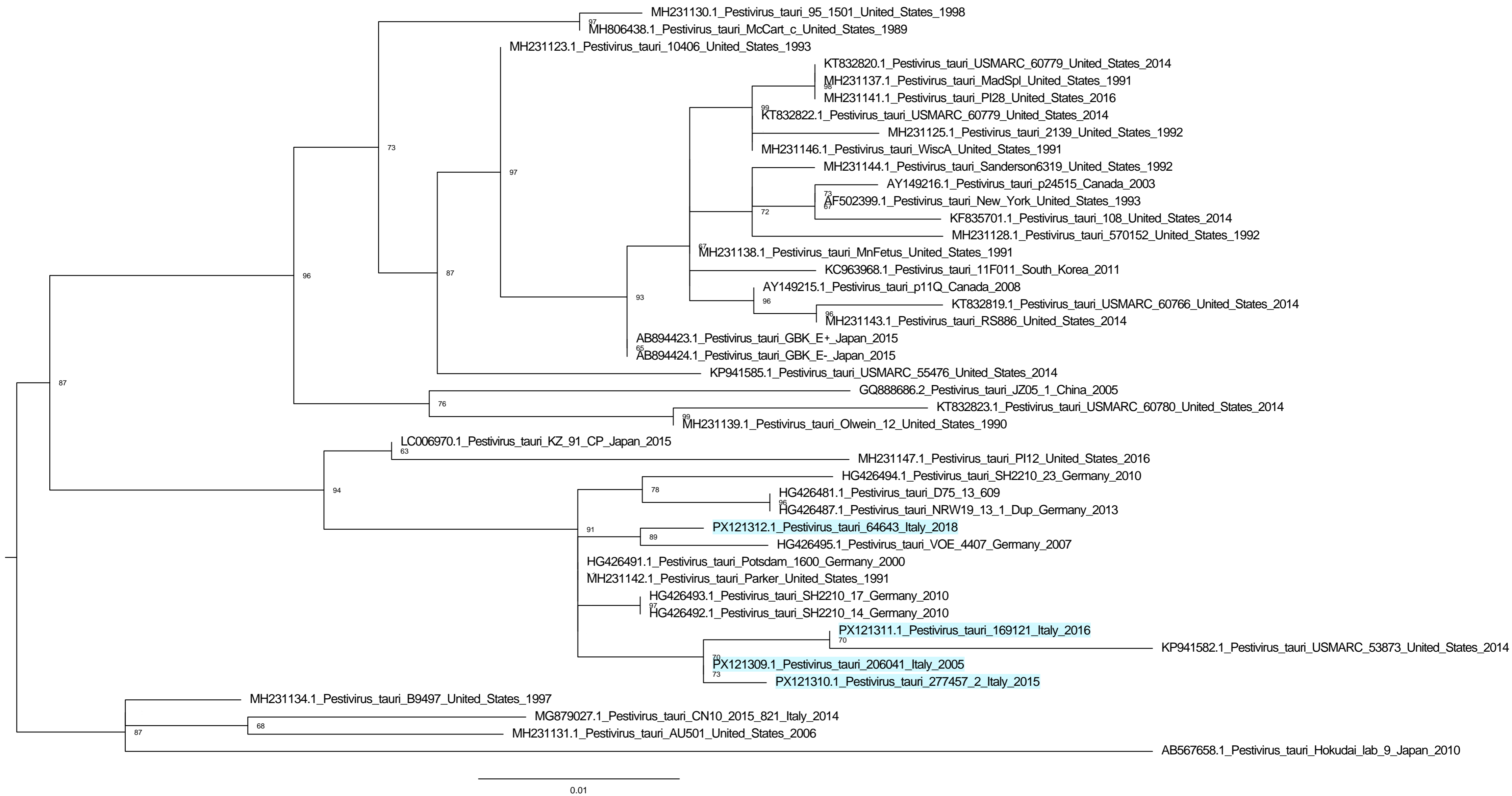

Supplement: Supplementary file 1 [file viruses-18-00367-s001.zip › FigureS1.pdf]

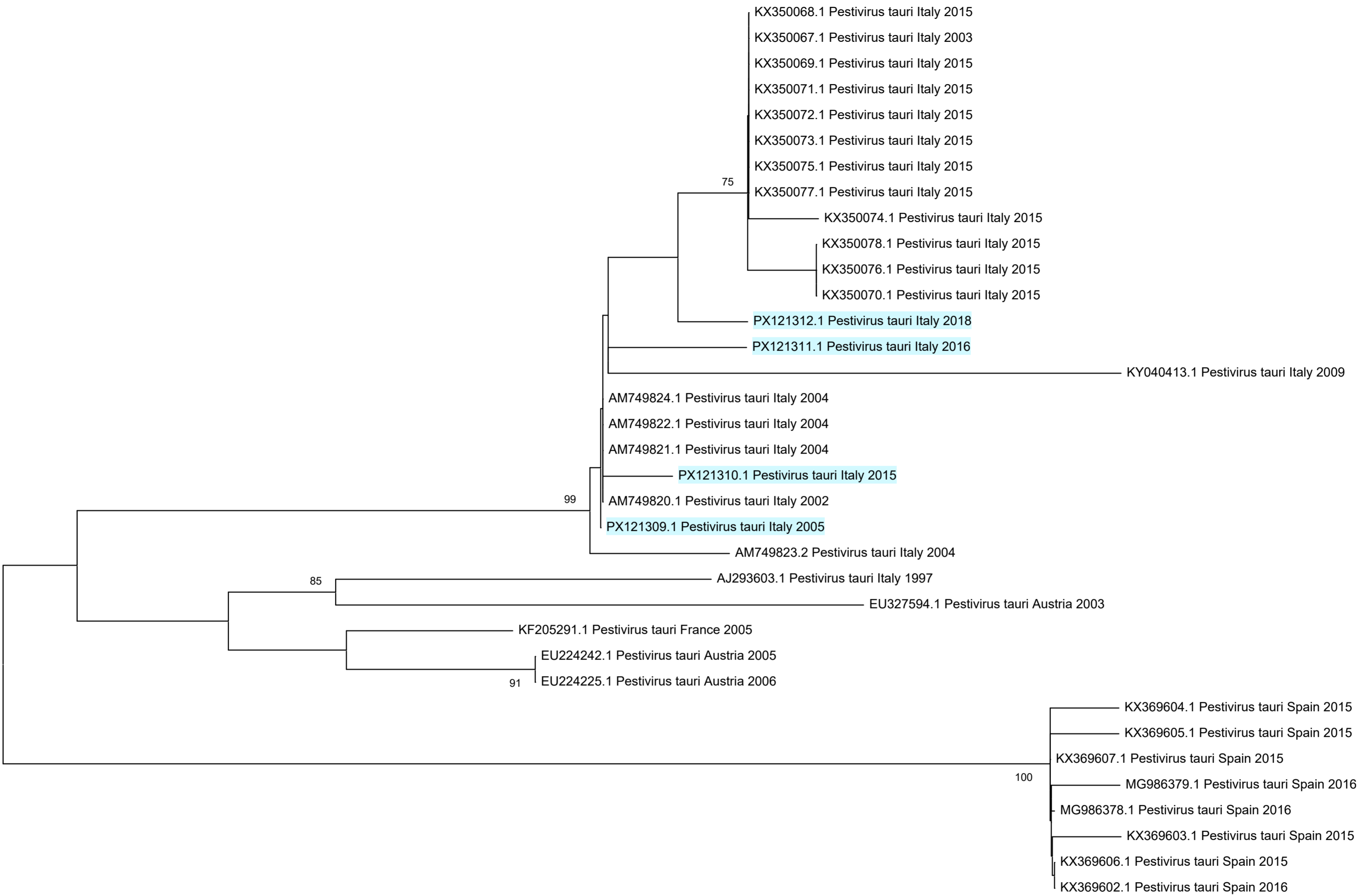

0.02

Supplement: Supplementary file 1 [file viruses-18-00367-s001.zip › FigureS2.pdf]
